# Airway mucus in infection

**DOI:** 10.3389/fphys.2026.1760997

**Published:** 2026-03-20

**Authors:** Caitlin Costello, Susan Birket

**Affiliations:** 1 Division of Pulmonary, Allergy, and Critical Care Medicine, Department of Medicine, University of Alabama at Birmingham, Birmingham, AL, United States; 2 Gregory Fleming James Cystic Fibrosis Research Center, University of Alabama at Birmingham, Birmingham, AL, United States

**Keywords:** airway, bacteria, epithelia, inflammation, mucus

## Abstract

The mucosa of the airways is under a near-constant barrage of contaminants, allergens, and pathogens that can accumulate and cause irritation or infection if not promptly removed. Mucus, composed of a mucin glycoprotein mesh, protects the airways from these contaminants by entrapment and removal. Several diseases stall the protection provided by the mucus by altering its components and biophysical properties. This review provides an overview of how the defensive mucus of the airways functions in health, how the mucus can fail to clear with muco-obstructive lung diseases, how mucins respond to pathogens that infect the mucus, how the molecular signals of mucin secretion function, and how therapeutics may improve morbidity and mortality for people with muco-obstructive lung disease. Recent studies of the spatial organization of mucin-producing cells have led to new understanding of the difference between constitutive MUC5B production by superficial epithelial cells and between trigger-induced production and secretion by the goblet cells and submucosal glands. The coordination of mucin production and secretion with ion and water homeostasis is discussed to evaluate how changes in sodium, calcium, bicarbonate, and chloride are responsible for failures of mucin unfolding and the subsequent biophysical properties of the mucus. Rheological and inflammatory characteristics of the muco-obstructive lung diseases are compared to determine how the defect in mucus clearance leads to distinct morbidity and mortality with attention paid to the microbial pathogens prevalent between groups. The distinctions between the molecular mechanisms of mucin production and secretion within muco-obstructive lung diseases are discussed with a focus on several cytokines such as IL-1β, secretagogues like ATP, and protein chaperones such as ERN2. Pathogenic induction of mucin production and secretion are discussed with primary focus on bacterial mediators. Finally, established and novel therapies for muco-obstructive lung diseases are discussed for potential at improving mucociliary clearance defects and for reducing exacerbation.

## Mucus in the airway

The mucosal surfaces of the airway come into contact and interact with the outside world in unique ways compared to other surfaces such as the skin. The mucosa are open to fragile areas of the body and so must handle the influx of debris and pathogens to prevent disruption of gas exchange. The key to this functionality is the presence of the eponymous mucus. Mucus is a hydrogel composed of 98% water, with mucins, DNA from sloughed host cells and lysed bacteria, globular host proteins, lipids, and host and pathogen cell debris comprising the remaining 2% (Potter et al., 1967; Yuan et al., 2015; Lethem et al., 1990; Lamblin et al., 1984). Mucins are glycoproteins composed of a protein backbone that is heavily O-glycosylated at the hydroxyl oxygen of the serine and threonine amino acids (Lamblin et al., 1984; Slayter et al., 1984; Rose et al., 1984). Mucins are high molecular weight due to the combination of polymerization of the mucin peptides and due to glycosylation adding many long, complex, branching chains of sugars to the peptide backbone (Thornton et al., 1990; Breg et al., 1987). The pattern of specific glycosylation for mucins has been investigated, finding a wide diversity of branching sugars with multiple terminal sugars (Lamblin et al., 1984; Slayter et al., 1984; Klein et al., 1988; Lamblin et al., 1991). Common terminal sugars are N-acetylneuraminic acid (sialic acid) and fucose with sulfation of these terminal sugars providing many combinations (Lamblin et al., 1984; Breg et al., 1987; Klein et al., 1988; Lamblin et al., 1991). The diversity of glycosylation present on mucins is vast and complex–depending on cell type, tissue type, and disease state (Barasch et al., 1991; Harris et al., 2024).

Inside the cell, mucins are densely packed into granules and can be secreted in consistent and continuous amounts to maintain homeostasis, as well as released in large quantities under stress stimuli such as from allergens or pathogens (Klinger et al., 1984; Neveu et al., 2009). When mucins are released into the airway, the proteins, which were tightly packed due to electrostatic interactions between the negatively-charged mucins, calcium, and hydrogen ions, begin to unfurl through replacement of the calcium with sodium ions and are swept along the epithelium by ciliary beating (Kesimer et al., 2010; Ermund et al., 2017; Ermund et al., 2018; Espinosa et al., 2002). Unfolding of mucins is dependent on concentrations of bicarbonate, calcium, and sodium in the airway with high concentrations of extracellular calcium, acidic pH, or hyperabsorption of sodium preventing unfolding of the gel-forming mucins (Kesimer et al., 2010; Espinosa et al., 2002; Livraghi-Butrico et al., 2013; Perez-Vilar et al., 2005; Garcia et al., 2009; Yang et al., 2013).

In the airways, the main mucin secreting bodies are submucosal glands containing mucous and serous cells, goblet cells, and club epithelial cells with expression of mucins being region-specific (Okuda et al., 2019). The primary secreters of homeostatic mucin are CCSP+ (SCGB1A1) club cells on the epithelia (Okuda et al., 2019; Hill et al., 2022). When responding to stress such as pathogen irritation or under mechanical induction via cough, goblet cells and submucosal glands release mucins from granules into the airway lumen. Submucosal glands typically secrete MUC5B, and goblet cells typically secrete MUC5AC in the proximal airways (Hovenberg et al., 1996; Groneberg et al., 2002). Superficial epithelial cells have also been found to strongly express MUC5B down into the distal bronchioles with significantly reduced MUC5AC in the distal airways overall (Okuda et al., 2019). In the terminal bronchioles, CCSP + club cells were not found to express either type of mucin and instead produced higher levels of surfactant proteins (Okuda et al., 2019). The smaller airways lead down into the alveoli where gas exchange occurs and where surfactants are increasingly required to prevent airway collapse during exhalation (Possmay et al., 2001).

When pathogens or other debris are trapped by mucus, ciliary beating pushes the mucus up the airways toward the trachea where the mucus is either swallowed and destroyed in the stomach or coughed out – a process termed mucociliary clearance (Iravani and Van As, 1972; Blake, 1975). Cilia act on mucus by moving in a metachronal waveform – an effective, or active, stroke that pushes mucus forward followed by a recovery stroke where the cilium slows down and curves away from the direction of the prior active stroke before beginning a new active stroke (Liu et al., 2014; Mitran, 2007). Without cilia to propel mucus up the airway, mucus would follow the force of gravity and pool in the lower airways – requiring the backup cough mechanism to clear (Bush et al., 2006; Möller et al., 2006). Cilia are capable of propelling mucus against gravity, but they are extremely sensitive to the viscosity and elasticity of the mucus (Blake, 1975; Liu et al., 2014; King, 1987; Tambascio et al., 2013; Girod et al., 1992). As viscosity is a measure of the ability of mucus to disperse energy and prevent flow, mucus that is too viscous can flatten the delicate cilia under the osmotic pressure and weight such that their waveform is ineffective or entirely blocked (Liu et al., 2014; Tambascio et al., 2013). High elasticity, as a measure of the ability of the mucus to store energy, can also prevent cilia from beating effectively by allowing a complete waveform but preventing sustained forward motion (Liu et al., 2014; Pino-Argumedo et al., 2022). It is important to note, however, that mucus with low viscosity would also be ineffective at clearing debris and pathogens, while mucus with low elasticity would prevent cilia from being able to propel the mucus forward. The rare case of bronchorrhea, where excess watery mucus is produced and typically causes prolonged cough, represents a case of mucus too thin for cilia to clear (Shimura et al., 1988; Bhagat et al., 2022; Rubin et al., 1991).

The other clearance mechanism available to the airway is coughing. The increased internal air pressure within the airway during a cough applies forces which can propel the mucus at high speed. Mucus can be sheared off the epithelium, determined by adhesive strength, or sheared apart from itself, determined by cohesive strength (Button et al., 2018). Importantly, viscosity and elasticity also play a role in the ability of the cough clearance mechanism to function. Mucus that is high in viscosity or elasticity may require higher than normal air pressure and shear forces to dislodge or tear apart and so may be resistant to cough clearance as well as ciliary clearance (King, 1987; Tambascio et al., 2013; Girod et al., 1992; Pillai et al., 1992).

Mucus acts both as a clearance mechanism through ciliary action and cough expulsion as well as a physical barrier blocking access to the epithelial layer. The dense, heavily branched nature of mucins helps block diffusion by creating a mesh with very fine pores (Button et al., 2012; Cone, 2009; Linssen et al., 2021). The upper layers of mucus containing secreted mucins (e.g., MUC5AC, MUC5B) block larger debris while the mucins associated with proximity to the epithelium, known as membrane-tethered mucins (e.g., MUC1, MUC4, MUC16), block access to the cilia and its underlying epithelium for debris down to 40 nm in size (Button et al., 2012; Kesimer et al., 2013). The glycosylation of the mucins also provides a physical and electrochemical barrier as the sugars interact with moieties on the surface of bacteria and viruses to bind and hold them in place – slowing diffusion rates and reducing the effectiveness of microbial motility (McAuley et al., 2017; Chatterjee et al., 2023; Fernández-Blanco et al., 2018). Tethered mucins help support the cilia during their metachronal wave pattern and against the osmotic pressure changes from water flowing between the mucus and epithelium (Button et al., 2012; Kesimer et al., 2013; Henderson et al., 2014). As with the physical trapping of debris within the sugars through physical and electrochemical interactions, host defenses released into the mucus can be immobilized by mucins as well. The airway and its resident immune cells produce antimicrobials (e.g., defensins) as well as immunoglobulins (e.g., IgG or sIgA) that can be effective at weakening, killing, or trapping bacteria, fungi, and viruses (Bals et al., 1998; Collin et al., 2020; Salathe et al., 2002). These antimicrobials and immunoglobulins can also be trapped in the mucus at the tethered mucin and secreted mucin layers, and this can be beneficial as it prevents the host defenses from being diffused away from the microbial targets. Another physical homeostasis function that mucus and its mucins perform in the airway is the maintenance of osmotic pressure and hydration (Girod et al., 1992; Henderson et al., 2014). The extensive glycosylation of the mucins means that they are highly hydrophilic and help to maintain the mucus as a gel-like fluid (Crouzier et al., 2015).

## Mucus in disease

As mucus is critical to defend the airways, dysfunction of the mucus layer can be long-lasting and detrimental to overall health (Konietzko, 1986). Dysfunction of the mucus often leads to plugging of the airways and chronic infection and thus are named muco-obstructive pulmonary diseases. Some common endotypes of these diseases, with similar presentations but differing etiologies, include cystic fibrosis (CF), chronic obstructive pulmonary disease (COPD), primary ciliary dyskinesia (PCD), and non-CF bronchiectasis (NCFB). Asthma, previously less associated with the other muco-obstructive lung diseases despite mucus plugging and infectious exacerbations, is now considered another endotype (Dunican et al., 2018a; Dunican et al., 2018b). The cumulative incidence of chronic respiratory disease is around 544.9 million people worldwide with COPD accounting for around 55% of the cases of chronic respiratory disease (GBD Chronic Respiratory Disease Collaborators, 2020). Asthma affects around 262 million people worldwide (WHO, 2024). However, CF, PCD, and NCFB are rarer with incidences ranging from 1 in 20,000 for PCD, between 50 and 1,000 in 100,000 for NCFB, and around 160,000 individuals with CF worldwide (Nigro et al., 2024; Guo et al., 2022; Kuehni et al., 2010). Chronic respiratory diseases are one of the leading causes of morbidity and mortality worldwide with many efforts focused on understanding the mechanisms underlying and treatments for these diseases. The mortality from muco-obstructive lung diseases comes from a combination of respiratory decline due to lung infection, persistent inflammation, airway damage, and obstructive mucus plugs (Goeminne et al., 2014; Courtney et al., 2007; Shah et al., 2016; Berry and Wise, 2010).

**FIGURE 1:**
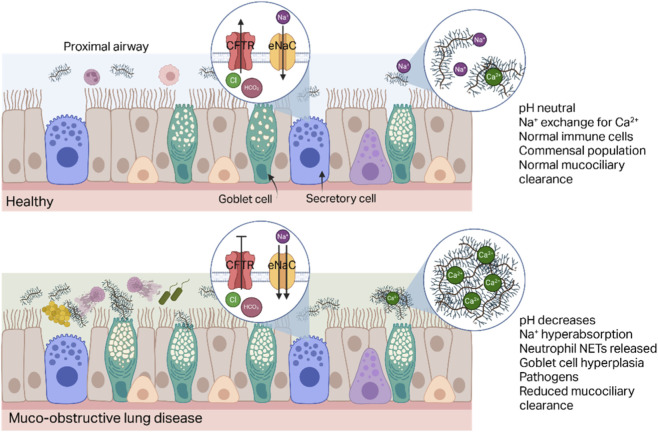
Schematic of selected differences in a healthy and a muco-obstructive proximal airway. The airway comprises ciliated, secretory, and goblet cells in the schematic. The goblet cells contain granules filled with mucin which are coiled around Ca^2+^ ions. As the mucin is released into the lumen, Na^+^ balance maintained by ion channels such as the epithelial Na channels (eNaC) replaces the Ca^2+^ to unfold the mucins. Water and pH balance is maintained by the flow of ions through channels such as the CFTR which secretes Cl^−^ and bicarbonate. Resident macrophages and neutrophils regulate the level of commensal bacteria. In muco-obstructive lung disease (bottom), dysfunction of ion channels such as CFTR can reduce ion flow – dropping the pH, increasing absorption of Na^+^, and insufficiently hydrating the mucus. Lack of water and Na^+^ in the lumen prevents untangling of the mucins during goblet cell secretion, reduces mucociliary clearance, and prevents pathogen clearance. Increased pathogen abundance activates neutrophils to produce NETs which accumulate cellular debris and DNA in the concentrated mucus and further frustrate clearance. Figure generated with Biorender.com.

Cystic fibrosis is one of the more well-understood chronic respiratory diseases due to its unique genetic background. CF is caused by recessive mutations in the cystic fibrosis transmembrane conductance regulator (*CFTR*) gene that then lead to incomplete transport to the membrane or malfunctioning of the CFTR protein (Van Goor et al., 2009; Welsh and Smith, 1993). CFTR is an ATP-controlled ion channel that moves chloride and bicarbonate ions out of the cell to help maintain hydration of the airway (Bear et al., 1992). Malfunctioning or missing CFTR leads to dysregulation of overall ion levels and hydration of the mucus. The failure of chloride and bicarbonate to be transported across the cell leads to increased sodium absorption and can prevent the exchange of calcium for sodium required to unfold mucins properly (Livraghi-Butrico et al., 2013; Boucher et al., 1988). Increased mucin secretion, flattening of the cilia under dehydrated, viscous mucus, and inflammatory responses are responsible for the mucus obstruction that occurs in CF (Figure 1). (Kato et al., 2022) As the mucins are secreted and insufficiently hydrated, the viscosity and elasticity of the mucus increase and make mucociliary and cough clearance more difficult (Tambascio et al., 2013; Batson et al., 2022; Ma et al., 2018; Tipirneni et al., 2018). The pathogens most commonly identified in people with CF change with age. Bacteria like *Haemophilus influenzae* and *Staphylococcus aureus* are more prevalent with younger people, and *Pseudomonas aeruginosa* is one of the most prevalent and most dangerous for older people with CF. (Cystic Fibrosis Foundation, 2021; Cystic Fibrosis Foundation, 2024; Ramphal, 1990; Schwab et al., 2014) Eradication is difficult for many of these pathogens due to the increased viscoelasticity of the mucus, antibiotic resistance of the bacteria, and antibiotic tolerance from biofilm formation of bacteria within the mucus (Singh et al., 2000; Landry et al., 2006; Greenwald et al., 2024; Wolter et al., 2013). The inflammation common to CF is typically neutrophilic whether sterile or induced from bacterial infection, resulting in release of DNA and granules via NETosis, a process of cell death through which neutrophil DNA and granule content “nets” are formed, and contributing to the non-productive muco-inflammatory cycle and the increased viscosity of the mucus (Linssen et al., 2021; Boboltz et al., 2023).

Primary ciliary dyskinesia is also caused by genetic mutations in over 40 different genes which result in the loss of ciliary function (Afzelius, 1976; Lucas et al., 2020). Loss of ciliary function can be from failure to produce cilia, loss of ciliary motor activity, failure to complete ciliary waveforms, and more (Möller et al., 2006; Lucas et al., 2020; Hornef et al., 2006). In a similar manner to CF, the inability to clear mucus through ciliary motion can induce a stress response which over-secretes mucin and leads to increased viscoelasticity of the mucus (Bush et al., 2006). Both CF and PCD result in mucus buildup and eventual plugging of the airways, decreasing respiratory efficiency and increasing likelihood of infection. As with CF, mutation types can affect severity, but all present with increased severity and bacterial infection status with advancing age (Wijers et al., 2017). PCD can be more severe at birth than CF due to the immediate failure of ciliary clearance, and so infants with PCD often present with more respiratory distress and develop bronchiectasis with recurrent infections earlier than those with CF (Möller et al., 2006; Halbeisen et al., 2018). PCD is similarly neutrophilic with neutrophil activity, neutrophil elastase, and IL-8 all increased in PCD patients (Bush et al., 2006). As a result, sputum viscoelastic properties are similar to CF sputum (Bush et al., 2006). A recent study by Nussstein et al. found that sputum rheological properties (elastic modulus, viscous modulus, and mesh pore size) for people with PCD were most similar to those of people with CF who had been on highly-effective modulator therapy elexacaftor/tezacaftor/ivacaftor (ETI) for 3 months (Nussstein et al., 2026). Additionally, proteomic investigation of key inflammatory markers such as DNA, myeloperoxidase, IL-1β, IL-8, and TNF-α were also more similar between the PCD and CF after ETI group compared to the higher levels of inflammatory markers for people with CF before ETI (Nussstein et al., 2026). Bronchiectasis, bronchial wall thickening, mucus plugging, atelectasis, and air trapping were all increased in PCD computed chest tomography (CT) scans with a tendency for worse presentation only in the middle and lower lobes when compared to CF scans (Tadd et al., 2019). The pathogens identified in PCD are similar to CF but with *H. influenzae* and *Streptococcus pneumoniae* more prevalent up to adolescence in people with PCD and *P. aeruginosa* beginning to dominate later in adolescence than in CF. (Shah et al., 2016; Wijers et al., 2017; Alanin et al., 2015)

Chronic obstructive pulmonary disease is typically acquired from prolonged damage to the airways through environmental exposures (Yoshida and Tuder, 2007). One of the most common factors leading to COPD is a history of smoking, although other particulate matter exposures, chemical exposures, respiratory infections, chronic bronchitis, asthma, and genetic susceptibility can also affect incidence (Yoshida and Tuder, 2007; Szalontai et al., 2021). COPD is split into two categories: emphysema and bronchitis (Yoshida and Tuder, 2007). While the emphysema state results in loss of alveolar spaces and low sputum production, the bronchitis phenotype results in narrowing of the airways as well as blocking of the alveolar spaces with mucus obstruction leading to chronic cough, sputum production, and exacerbations (Szalontai et al., 2021; Flenley, 1988; Lin et al., 2020). The main pathogens isolated from people with COPD include *H. influenzae*, *S. pneumoniae, Moraxella catarrhalis*, and *P. aeruginosa* (Miravitlles et al., 1999; Groenewegen et al., 2003; Ko et al., 2007; Garcia-Vidal et al., 2009; Sibila et al., 2016). Infection with *P. aeruginosa* is lower than other chronic respiratory diseases (∼4–20%) but increases the risk of exacerbations and mortality in people with COPD (Eklöf et al., 2020). Increased neutrophilic inflammation and extracellular DNA from sloughed epithelial cells and NETs are also common in COPD and have been correlated with COPD pathology regardless of infection-status or smoking status (Pedersen et al., 2015). Murine models treating elastase generate COPD-like mucus obstruction from goblet cell hyperplasia, inflammatory responses, and emphysema (Fernández-Blanco et al., 2018).

Non-cystic fibrosis bronchiectasis generally presents with dilated airways, excess mucus secretion, persistent cough, and frequent respiratory infections in similar patterns as other muco-obstructive pulmonary diseases (Ramsey et al., 2020; Keir and Chalmers, 2021; McShane et al., 2013). Acquisition of NCFB can be idiopathic but is associated with increased age (McShane et al., 2013). While no cause has been identified, tuberculosis or *Aspergillus fumigatus* infection, asthma, immune deficiencies, pneumonia, and recurrent aspirations are posited as potential associations with development of NCFB (Nigro et al., 2024; McShane et al., 2013; Pasteur et al., 2010; Kapur et al., 2012; Lonni et al., 2015). A study of pediatric patients newly diagnosed with NCFB found that *H. influenzae*, *S. pneumoniae*, and *S. aureus* were among the most common pathogens isolated from the lower airway (Kapur et al., 2012). *P. aeruginosa* was only present in a small proportion of this population, but those with *P. aeruginosa* infection were more likely to have co-morbidities. Like CF and PCD, neutrophilic inflammation was prevalent and associated with higher overall inflammation and respiratory infection (Kapur et al., 2012). Just as incidence of *P. aeruginosa* increases with age for CF and PCD, people with NCFB are more likely to acquire and maintain chronic infection with *P. aeruginosa* as they age, and this association has been linked to higher morbidity and mortality than other pathogens common to NCFB infection (Loebinger et al., 2009). People with NCFB can also present with COPD or asthma, and those who do tend to experience higher levels of exacerbation and mortality (Goeminne et al., 2014; Keistinen et al., 1997).

Asthma is acquired through a combination of genetic and environmental factors (Beasley et al., 2000; Resiliac and Grayson, 2019; Thomsen, 2015). Asthma presents with mucus obstruction, hyperreactivity, cough, wheezing, and shortness of breath (Beasley et al., 2000; Resiliac and Grayson, 2019). Hyperreactivity or asthma attacks are frequently the cause of death for people with asthma due to oxygen deprivation and respiratory distress from mucus obstruction (Yoshida et al., 2020). Infections with asthma tend to be more viral than bacterial (Papadopoulos et al., 2011), although a few bacterial species (e.g., *S*. *pneumoniae, H. influenzae, Chlamydia pneumoniae* and *Mycoplasma pneumoniae*) can be found in chronic infections in the asthmatic airway (Iikura et al., 2015). Respiratory syncytial virus (RSV), rhinoviruses, influenza viruses, and more can exacerbate wheezing, inflammatory responses, and subsequent airway remodeling (Iikura et al., 2015; Taussig et al., 2003). In comparison to the other muco-obstructive pulmonary diseases where neutrophils predominate, asthma inflammatory responses are dominated by eosinophils (Dunican et al., 2018a; Carr et al., 2016; Chan et al., 2023; Liegeois et al., 2025). Eosinophils are typically responsible for destroying parasites or viruses through release of reactive oxygen species from granules (Ravin and Loy, 2016). Eosinophil granule contents, such as major basic protein (MBP), play a large role in the hyperreactive and pro-inflammatory airway of asthma as the contents are toxic to both pathogens and host cells, and higher eosinophil counts are related to frequency of exacerbations (Dunican et al., 2018a; Carr et al., 2016; Green et al., 2002). Airway epithelial cells cocultured with eosinophils increased MUC5AC secretion directly in an amphiregulin- and TGF-α-dependent mechanism (Shimizu et al., 2014). Increased mucin secretion, particularly MUC5AC, is associated with asthma, and the mucus plugs generated by the asthmatic airway are hypothesized to be generated by eosinophil peroxidase crosslinking of mucus thiol groups to stiffen the mucus gels and prevent clearance (Dunican et al., 2018a). This is similar to work hypothesizing that the increased oxidative stress from neutrophil NETs and elastase increases mucus stiffness in CF and COPD (Uddin et al., 2019).

Across these diseases, the importance of mucus in airway defense is clear. The property of mucus that determines its transportability is its viscoelasticity (Ma et al., 2018; Nielsen et al., 2004). Viscoelasticity is a measurement of how substances flow and resist force (Lai et al., 2009). Plugs are typically associated with the smaller bronchioles due to their small diameter where the shear force from coughing is reduced overall and adhesion of the mucus to the walls of the airways can block airflow (Chan et al., 2023; Panchabhai et al., 2016; Langlands, 1967; Hasani et al., 1994). Mucus plugs can prevent gas exchange and are often niches for infectious microorganisms to evade immune defenses and chronically infect. There is also a vicious inflammatory cycle that persists in muco-obstructive lung disease where mucin hypersecretion and insufficient hydration lower oxygen availability (hypoxia), triggering inflammatory responses that further increase mucin secretion to attempt to clear the trapped mucus (Worlitzsch et al., 2002; Fri et al., 2015; Cowley et al., 2015). Neutrophils are signaled to the areas of inflammation even without infection present, but bacterial presence can push this neutrophilic response further, generating more oxidative stress from released reactive oxygen species, more crosslinking of the mucins from the oxidative stress, and more DNA released from dying epithelial and neutrophil cells (Yuan et al., 2015; Lethem et al., 1990; Batson et al., 2022; Pedersen et al., 2015; Saffarzadeh et al., 2012; Liu et al., 2024). All these inflammatory effects compound and make clearing those areas of obstructed mucus challenging for the mucociliary apparatus.

## Molecular mechanisms of increased mucus secretion

Secretion of mucins in the airway is continuous, with induced increases in mucin production intracellularly correlating to more concentrated mucin secretion, but secretagogues, such as ATP or histamine, are responsible for the rapid, complete release of granule contents accumulated within mucin-producing cells into the airway lumen (Jaramillo et al., 2018). Mucous cell (i.e., goblet, epithelial) meta- or hyperplasia in combination with the accumulation of mucin protein within mucous cells generate the quantities of mucin which create obstructions. The main pathway involved in the regulation of mucus production within muco-obstructive lung disease is the interleukin-1 (IL-1) family of cytokines and their downstream effectors. IL-1β and IL-1α have been strongly associated with increased MUC5B and MUC5AC production and secretion (Chen et al., 2019; Sponchiado et al., 2023; Fujisawa et al., 2011; Lappalainen et al., 2005). These cytokines bind to IL-1R and can induce activity of SAM pointed domain containing ETS transcription factor (SPDEF) and other downstream regulators like anterior gradient 2 (AGR2) and endoplasmic reticulum to nucleus signaling 2 protein (ERN2) which mediate mucin folding (Chen et al., 2019; Sponchiado et al., 2023; Cloots et al., 2024). ERN2 and AGR2 are endoplasmic reticulum stress response proteins that manage gene expression of MUC5AC and MUC5B, and AGR2 also acts as a chaperone for mucin proteins to ensure proper folding (Chen et al., 2019; Cloots et al., 2024). IL-13, another cytokine known to induce mucin production, has generally been associated with the upregulation of MUC5AC instead of MUC5B in human bronchial epithelial cells but has been shown to upregulate both MUC5AC and MUC5B in murine models (Zhen et al., 2007; Sponchiado et al., 2024; Whittaker et al., 2002). As asthma generally presents with a high MUC5AC to MUC5B ratio and high IL-13, IL-13 has been hypothesized as responsible for the increased MUC5AC concentration in asthmatic patients (Liegeois et al., 2025; Lachowicz-Scroggins et al., 2016). IL-1β, IL-1α, and IL-13 are also involved in mucous cell differentiation with increased expression of these cytokines leading to mucous cell metaplasia (Lappalainen et al., 2005; Zhen et al., 2007). Silencing RNA (siRNA) knockdown of Muc5b was shown to decrease IL-1β levels but did not alter IL-1α levels in a *CFTR* knockout rat model infected with *P. aeruginosa* after 2 weeks of infection compared to rats given siRNA unrelated to Muc5b (Murphree-Terry et al., 2024). Knockdown of one factor in the pathway may improve but not fully disrupt the muco-inflammatory cycle due to the multifactorial and overlapping regulators of mucin secretion.

Additional cytokines and growth factors that are involved in mucin expression and secretion include IL-8 (Bautista et al., 2009), IL-17A (Fujisawa et al., 2011; Xia et al., 2014), IL-6 (Neveu et al., 2009; Chen et al., 2003), TNF-α (Fischer et al., 1999; Lora et al., 2005; Busse et al., 2005), TGF-α (Shao et al., 2003; Baginski et al., 2006), and EGFR (Zhen et al., 2007; Shao et al., 2003; Nadel and Burgel, 2001). IL-8 has been shown to act on mucin post-translationally through increasing the levels of RNA-binding proteins to improve stability of the mucin transcripts (Bautista et al., 2009). IL-17A and IL-6 caused an increase in both *MUC5B* and *MUC5AC* gene expression in primary tracheal epithelial cells (Chen et al., 2003), and IL-17A caused an increase of MUC5AC in primary human bronchial epithelial cells through the NF-κB enhancer of MUC5AC gene transcription (Fujisawa et al., 2011; Xia et al., 2014). Some cytokines, such as TNF-α and TGF-α, increase mucin secretion through the interaction of the EGFR and reactive oxygen species pathways as well as generation of small nucleotides which trigger mucin granule exocytosis such as cyclic-di-GMP, ATP, or cAMP (Perez-Vilar et al., 2005; Fischer et al., 1999; Okada et al., 2011). TNF-α increases mucin secretion through the production of nitric oxide and cyclic-di-GMP, induction of the NF-κB pathway, and goblet cell metaplasia in mice (Fischer et al., 1999; Busse et al., 2005). Pyocyanin produced by *P. aeruginosa* has been shown to upregulate mucin secretion through the activation of the EGFR pathway by inducing intracellular reactive oxygen species, triggering cytokine production (e.g., IL-1β, IL-1α, IL-6, IL-8, TNF-α), and generating EGFR ligands such as TGF-α in epithelial cells (Shao et al., 2003; Nadel and Burgel, 2001; Rada et al., 2011).

## Mucus in response to infection

Responding to an infection is a balance between destruction and removal of the pathogen and survival of the host cells. In the lungs, mucus is constantly secreted by epithelial, goblet cells, and submucosal glands to maintain clear and clean airways (Ermund et al., 2017; Ermund et al., 2018; Ermund et al., 2021). When pathogens are detected, through mechanisms like pathogen-associated molecular patterns (PAMPs), damage-associated molecular patterns (DAMPs), or through detection of pathogen toxins, mucus-producing cells can dramatically increase mucin production and secretion (Rada et al., 2011; Smirnova et al., 2003). Submucosal glands are particularly known to release their vesicular contents when triggered by pathogens (Ermund et al., 2017), and the mucin bundles expand from their densely packed, folded state to sweep up and clear pathogens out. Commensal bacteria which colonize the lower respiratory tract and maintain low bacterial density are similar to those of the mouth and upper respiratory tract, likely due to repeated microaspirations of the saliva and nasal mucus into the trachea (Segal et al., 2016). These bacteria have been shown to induce Th17 and neutrophilic responses in the lung mucosa and inhibit TLR4 and eosinophilic responses and so may be important in the tuning and sensitivity of the respiratory mucosal immune response (Segal et al., 2016; Mathieu et al., 2018). With muco-obstructive lung disease, the failure of mucociliary clearance mechanisms and the shifting of metabolic environments provide a niche for opportunistic bacterial pathogens to overwhelm the existing commensal microbiota.

Gram-negative bacteria, like *P*. *aeruginosa* or *H. influenzae*, have several PAMPs and toxins which stimulate inflammatory responses. Lipopolysaccharide (LPS) on the outer membrane of Gram-negatives are a common PAMP known to induce inflammatory responses (e.g., IL-8) and subsequently inducing mucin secretion (e.g., MUC5AC, MUC5B) (Smirnova et al., 2003; Dohrman et al., 1998; Widdicombe, 1995). The flagellum produced by *P. aeruginosa* has also been shown to increase inflammatory and mucin secretion responses (Mohamed et al., 2012). Pyocyanin, a virulence factor produced by *P. aeruginosa*, is a powerful reactive oxygen species producer that can kill both competing bacteria and nearby host cells, and it has been shown to initiate stress responses in ciliated epithelial cells such as triggering increased ciliary beat frequency at low doses, decreased ciliary beat frequency at high doses, and increased mucin secretion (Rada et al., 2011; Ka et al., 1993). Enzymes produced by *P. aeruginosa*, alkaline proteinase and elastase, both cause a dose-dependent increase in mucin secretion on *ex-vivo* rabbit trachea (Klinger et al., 1984; Everett et al., 2023). Our lab has also shown that *P. aeruginosa* is capable of altering mucus viscoelasticity and transportability through production of a mucinase (Costello et al., 2025). Similarly, the fimbriae of *H. influenzae* contribute to mucus binding properties, and in a study of *H. influenzae* attachment, fimbriated strains were localized on the epithelium only when the cell layer was damaged (Barsum et al., 1995). Lysates of nontypeable *H. influenzae*, containing cytoplasmic proteins that would be released during antibiotic treatment or host-mediated lysis, were shown to induce MUC5AC gene expression in human lung epithelial cells (Wang et al., 2002). Lipoprotein P6, on the outer membrane of *H. influenzae*, was also found to induce MUC5AC gene expression in human lung epithelial cells and in mice trachea treated with P6 (Chen et al., 2004). Interestingly, mucin is also capable of regulation of bacterial functions; works by Wang et al. and Wheeler et al. have found that *P. aeruginosa* senses the glycans present on mucins and decreases virulence of *P. aeruginosa* against other bacteria through the inhibition of the type VI secretion system (Wheeler et al., 2019; Wang et al., 2021). This decreased virulence is suggested by the authors to partially explain why *P. aeruginosa* does not predominate in a healthy airway, and shifts in the mucin glycosylation patterns or accessibility of the glycans to the sensor are suggested as possibilities for the dominance of *P. aeruginosa* in CF. (Wang et al., 2021)

These effects are not limited to Gram-negatives, as cell-free conditioned media from *Streptococcus pneumoniae*, a Gram-positive bacterium, has also been shown to induce mucin secretion (Adler et al., 1986). While the mechanism through which *S. pneumoniae* cell-free conditioned media increases mucin secretion has not been identified, *S. pneumoniae* has been shown to use neuraminidase, an enzyme that cleaves sialic acid off mucins, to alter the mucus layer to allow closer contact with the epithelium (Widdicombe, 1995; Montgomery et al., 2025). *S. pneumoniae* has also been shown to be able to cleave MUC16 from tracheobronchial cells using a metalloproteinase ZmpC (Govindarajan et al., 2012). *Staphylococcus aureus*, one of the earliest and most persistent pathogens found in the lungs of people with CF, has been shown to adapt to the lung environment by increasing pro-inflammatory cytokine secretion (e.g., TNF-α or IL-8, involved in the mucin secretion pathway) through upregulating staphylococcal protein A (Spa) (Ramond et al., 2022). The Spl protein of *S. aureus* has also been shown to be involved in spreading of the bacteria into both lobes of rabbit lungs and can cleave the tethered MUC16 off CALU3 epithelial cells (Paharik et al., 2016). One group described an increase in MUC5AC and MUC2 gene expression in human epithelial cells exposed to *S. aureus* cell-free supernatants (Dohrman et al., 1998), although no direct mechanism for *S. aureus* to increase mucin secretion has been described. The mechanisms the airways use to detect bacterial pathogens and their toxins are numerous and often non-specific. Recognition of pathogen presence can yield several responses, including increased mucin secretion, increased ciliary beat frequency, and pro-inflammatory signaling if bacteria are not cleared effectively by the former actions. Alternatively, some pathogens may be able to mimic host regulatory pathways to evade or dampen immune responses. One such example of this is the activity of the capsular polysaccharide produced by Group B *Streptococcus* (GBS); the bacterium caps its sugars with a terminal sialic acid, which is recognized by the lectin Siglec-9 on neutrophils as an inhibitory signal, thus dampening the ability of the neutrophils to kill the bacteria (Carlin et al., 2009; Chang et al., 2014).

Both viral and fungal pathogens may also increase mucin secretion. Rhinovirus (RV) and RSV have both been shown to increase MUC5AC secretion through epidermal growth factor receptor (EGFR) and extracellular ATP release (Okada et al., 2011; Singanayagam et al., 2022; Hewson et al., 2010). People infected with the *hanks* type rhinovirus for 5 days were shown to have significant increases in mucin secretion determined by alcian blue staining in nasal lavages (Yuta et al., 1998). Another study done by infecting both asthmatic and non-asthmatic people with RV-16 found that RV increases MUC5AC protein secretion and transcription over 4 days of infection. Peaks in protein levels were seen for non-asthmatic people at 4 days post infection, but the asthmatic people had higher baseline and post-infection protein levels in their bronchial lavage and bronchial biopsies than the non-asthmatic group indicating deficits in resolution of the increased mucin secretion (Hewson et al., 2010). RSV infection increased MUC5AC gene expression and protein production in human bronchial epithelial cells (Chi et al., 2022), and a chimera study done between the virulent RSV line 19 strain and less virulent RSV A2 laboratory strain found that the fusion protein increased IL-13 levels and mucin secretion in mice when present in the less virulent strain (Moore et al., 2009).

Just as bacterial proteinases and toxins can increase pro-inflammatory mucin secretion, so too can fungi. Work done with *Aspergillus fumigatus*, a fungus that causes Aspergillosis and has been associated with acute respiratory distress syndrome (ARDS) during the COVID-19 pandemic, showed that *A. fumigatus* increased MUC5AC protein expression and increased the activity of tumor necrosis factor-α-converting enzyme (TACE) through serine protease cleavage (Oguma et al., 2011). Mucus concentration has been shown to impact *A. fumigatus* with higher concentrations of mucin increasing growth and dilutions of the mucin decreasing growth (Poore et al., 2025).

## Therapeutics

Maintaining a healthy level of mucus is necessary for defense against pathogens and other debris in the airways, but as this review has discussed, there are several diseases where the mucin concentration is elevated and detrimental to health. Treatments for muco-obstructive lung diseases do exist, and these treatments have vastly improved the lives of some people living with these diseases (Table 1). There is no treatment that is completely effective at removing all impacts on mucus and infection, however, and so research continues on several different classes of therapeutics targeting mucus and its pathogens.

**TABLE 1:** Therapeutics improving airway mucus accumulation.

Therapeutic	Class	Year of FDA-approval	Direct or indirect mucus clearance	References
Hypertonic saline	Mucus thinner	2014	Direct	Donaldson et al. (2006), Elkins et al. (2006), Bennett et al. (2015), Paff et al. (2017)
rhDNase (Pulmozyme)	Muco-lytic	1990	Direct	Fuchs et al. (1994), Daiya and Sierra (2023)
N-acetylcysteine	Muco-lytic	1963	Direct	Batson et al. (2022), Conrad et al. (2015)
Carbocysteine	Muco-lytic	NA	Direct	Minov et al. (2019)
Elexacaftor/tezacaftor/ivacaftor (Trikafta)	CFTR modulator	2019	Indirect	Donaldson et al. (2024), Nichols et al. (2022)
Denufosol	P2Y purinoceptor agonist	NA	Indirect	Accurso et al. (2011), Ratjen et al. (2012)
Duramycin	Calcium activated chloride channel agonist	Off-label	Indirect	Oliynyk et al. (2010)

Many mucus-targeting therapeutics act by rehydrating and cleaving mucins. One of the largest breakthroughs in treating CF was the implementation of ivacaftor, a CFTR potentiator, which increases CFTR activity by improving the ion channel gating (Van Goor et al., 2009). This rescues airway hydration and has allowed mucus clearance to be restored. People with CF on ivacaftor experienced fewer exacerbation events requiring antibiotics and hospitalization (Rowe et al., 2014; Rowe et al., 2017; Ramsey et al., 2011). The approval of the therapy combining ivacaftor, elexacaftor, and tezacaftor provides expanded treatment options for those with misfolded CFTR proteins as well since elexacaftor and tezacaftor improve the processing and trafficking of CFTR (Donaldson et al., 2024; Nichols et al., 2022; Rowe et al., 2017; Veit et al., 2021). A clinical study following people with CF on elexacaftor/tezacaftor/ivacaftor (ETI) for 6 months found a significant reduction in antibiotic use by the end of the study–suggesting a lower prevalence of bacterial-induced exacerbations (Nichols et al., 2022). An additional clinical study of people beginning ETI found significant improvements in mucociliary clearance by radioactive isotope tracking (Donaldson et al., 2024). Even with the use of highly effective modulator therapies like ETI, bacterial infection remains a significant concern for people with CF (Cystic Fibrosis Foundation, 2024). Other methods of increasing mucus hydration have been direct-acting, such as hypertonic saline (HTS). HTS is nebulized to rehydrate mucus through altering osmotic pressure. This method can work, but the full efficacy appears to be limited to during and shortly after the time of nebulization (Bennett et al., 2015). While it can be beneficial, HTS has only minor efficacy for CF and even less for NCFB and PCD (Donaldson et al., 2006; Elkins et al., 2006; Paff et al., 2017). For infectious outcomes, treatment with HTS was shown to reduce exacerbations overall for people with CF, but it did not alter bacterial burden for *S. aureus* or *P. aeruginosa* (Elkins et al., 2006). Along the same lines as ivacaftor and hypertonic saline, several pharmaceutical groups have been pursuing pharmaceuticals that target other ion channels to improve mucociliary clearance in a *cftr*-independent manner. For example, pharmaceuticals such as ETX001 (Danahay H. L. et al., 2020), E_ACT_ or F_ACT_ (Namkung et al., 2011) which would increase the activity of TMEM16A, subsequently increasing chloride and bicarbonate transport to propel water into mucus, or decrease the activity of ENaC, reducing sodium transport intracellularly and increasing water retained in mucus, could be very beneficial but are still far from public use (Livraghi-Butrico et al., 2013; Danahay H. L. et al., 2020; Al-Hosni et al., 2022; Danahay et al., 2024; Danahay H. et al., 2020). P2Y_2_ receptor agonists, denufosol (Accurso et al., 2011) and duramycin (OLIYNYK et al., 2010), were promising for their ability to activate alternative chloride secretion pathways, but both of these agonists have not been pursued further due to not meeting primary study goals of improving FEV_1_ (Accurso et al., 2011; Ratjen et al., 2012) and having a narrow effective concentration range, respectively (OLIYNYK et al., 2010).

Mucolytics, or mucus-cleaving agents, are similar to the rehydrating efforts of hypertonic saline but with the potential for more long-lasting benefits. Recombinant human dornase alfa (rhDNase) has been used to break down the excess DNA present within the airway mucus for people with CF and has been somewhat successful at reducing viscoelasticity of mucus and improving clearance, but DNase has not proven beneficial for people with NCFB (Pasteur et al., 2010; Fuchs et al., 1994; Patarin et al., 2020). Due to the high number of disulfide bonds between mucus strands in the concentrated state, efforts to reduce these bonds and untangle mucins have involved the use of N-acetylcysteine, carbocysteine, and new long-lasting reducing agents such as P3001 or MUC-031 (Batson et al., 2022; Ehre et al., 2019; Addante et al., 2023). These agents act to break apart the mucus bundles from their compacted, tangled state to reduce viscoelasticity and improve clearance, but they are still in the early stages of testing to determine how effective the new mucolytics are compared to the older N-acetylcysteine and carbocysteine. N-acetylcysteine treatment has been shown *in vitro* to improve RSV outcomes in mucin hypersecretion and viral load (Chi et al., 2022). In a clinical trial where people with CF were given N-acetylcysteine for 24 weeks and followed for lung function and pulmonary exacerbations, N-acetylcysteine maintained baseline lung function longer than the placebo group but failed to show a significant improvement in exacerbations (Conrad et al., 2015). In contrast, a study following people with bronchiectasis given carbocysteine for 3 months found reduced exacerbation frequency and duration (Minov et al., 2019).

Attempts to modify the molecular basis behind increased mucin secretion have also been pursued but with limited success. EGFR antagonists are examples of efforts to modify goblet cell formation and subsequent mucin secretion, but none have progressed. The EGFR antagonists, BIBX 1382 BS (Dittrich et al., 2002) and BIBW 2948 (Woodruff et al., 2010), had limited efficacy and low tolerance by the people using the agent. For people with COPD, long-acting muscarinic antagonists (LAMA), which act as bronchodilators, have also been shown to reduce mucus secretion (Ramnarine et al., 1996; Calzetta et al., 2022; Mullol et al., 1992). The LAMAs, while generally proposed to act on smooth muscle, may also act on mucus-producing epithelial cells, submucosal glands, goblet cells, and even on the inflammatory cells that contribute to the muco-inflammatory cycle. People on LAMAs with COPD and asthma have been reported to improve sputum and cough expectoration and so may also have reduced mucus production (Calzetta et al., 2022), but these bronchodilators have not been studied sufficiently to show a strong effect for people with CF, NCFB, or PCD (Smith and Edwards, 2017; Ratjen et al., 2015; Pollak et al., 2025; Balfour-Lynn et al., 2006).

## Conclusion

Even with the existing mucus hydrators, mucolytics, and antibiotics, mucus hyperconcentration and bacterial infection remain significant areas of concern for people with muco-obstructive lung disease. The human airway receives many insults from pathogens, pollen, dust, etc., every day that can contribute to illness, and people who have predispositions to deficiencies in mucus clearance are far more likely to experience an insult that tips their airway from maintaining homeostasis to cycling indefinitely between mucus hypersecretion, dehydration, plugging, and infection. These problems have been improved for people with certain *cftr* mutations with the advent of *cftr* modulators, but the other muco-obstructive diseases still have far to go for therapies that will restore mucus back to its protective state. Key questions remain regarding the mechanisms behind muco-obstructive lung disease and the infections which seize upon the deficient clearance. Does the lung microbiota contribute to the risk of developing or the severity of muco-obstructive lung disease? What factors of the clearance defect (e.g., mucin, ciliary, immune) determine which pathogen predominates in the airway? Is remediation of the mucociliary clearance defect sufficient to reduce severe exacerbation? Muco-obstructive lung diseases remain an area of continuing research, with improvements in glycomics, metabolomics, genomics, and microbiomics providing new methods of investigating these questions for targeted therapeutic development.
